# Supplementary material for: Are carbon emissions trading and green financial instruments synergistic? -Comprehensive quantitative research based on content analysis
Source: PLoS One. 2024 Mar 7;19(3):e0298601. doi: 10.1371/journal.pone.0298601 (PMC10919601; doi:10.1371/journal.pone.0298601)
Supplement: S1 Data — (ZIP) [file pone.0298601.s001.zip › Data/地级市Z_F工作报告.zip!/╣π╢1⁄2/╔╟╬▓╩╨/└┤╫╘╣1⁄2╓┌║┼í╛┬φ┐╦╩2╛▌═°í┐_2007.docx]

各位代表：

我代表市人民政府向大会作政府工作报告，请予审议，并请政协各位委员和其他列席人员提出意见。

2004至2006年工作回顾

　　第四届市政府于2004年7月换届产生。近三年来，在省委省政府和市委的正确领导下，在市人大和市政协的监督和支持下，我们紧紧围绕"全面推进奔小康，再造一个新汕尾"的总目标，坚持"一手抓发展，一手抓稳定"，促进了国民经济持续、快速、健康发展，政治文明、精神文明和社会各项事业都取得了可喜的成绩。主要体现在十个方面：

(一)经济增长逐年提速，经济实力明显增强。2006年全市完成地区生产总值247.5亿元，比上年增长15.8％，比2003年增长55.1％，年均增长15.8％；工业总产值379亿元，比上年增长26.1％，比2003年增长101％，年均增长26.2％；固定资产投资134.3亿元，比上年增长31％，比2003年增长135.8％，年均增长33.1％：外商直接投资1.45亿美元，比上年增长19.9％，比2003年增长90％，年均增长23.9％；外贸出口总额8.54亿美元，比上年增长33.1％，比2003年增长220.9％，年均增长47.5％；地方财政一般预算收入9.27亿元，比上年增长30.7％，比2003年增长97.4％，年均增长25.4％；社会消费品零售总额153.94亿元，比上年增长18.1％，比2003年增长56.5％，年均增长16.1％。三次产业结构逐年优化，由2003年的31.9:32.2:35.9调整为22.4:41.9:35.7。

(二)工业化进程明显加快，经济效益不断提高。工业经济总量快速扩张，工业在国民经济中的地位不断增强。2006年工业增加值占GDP的比重为36.1％，比2003年提高9.9个百分点。以信利、德昌电子信息产业为龙头的规模以上工业发展加快，工业效益稳步提高。2006年全市规模以上工业经济效益综合指数达152％，创历史最高水平。企业自主创新能力逐步增强，全市现有国家免检产品5个，省著名商标7个，省名牌产品4个。

(三)基础设施建设不断加强，海滨城市初具规模。基础设施和重点项目建设稳步推进，固定资产投资不断扩大。三年累计完成固定资产投资310.5亿元，年均增长33.1％。三年来，全市共投入资金256.85亿元，用于交通、通讯、能源、水利、市政等基础设施建设。其中交通建设投入资金近20亿元，新(改)建公路里程1343公里。总投资2亿多元的500千伏汕尾茅湖变电站工程已顺利竣工。汕尾发电厂(一期)已投入资金43亿元，完成工程总进度87.5％。市区"三区六路一广场"工程建设顺利推进。凤山妈祖文化广场一期工程、玉台山慈云公园、汕长一级公路、市区至海城一级公路等基本建成；市区海滨大道二期工程、奎山河综合改造工程、全民健身广场等市政十大工程已投入使用，尤其是环品清湖沿岸的集市区防洪、市政道路、城市景观为一体的综合开发项目已完成海堤建设4.3公里，城市空间进一步拓展，城市品位明显提升。城市规划建设管理得到加强，市区市容明显改善，城市面貌焕然一新，适宜居住、创业、休闲的现代化滨海新城已初具规模。

(四)对外开放继续扩大，利用外资成效显著。三年来，累计完成外贸出口总额19亿美元，年均增长47.5％；累计利用外商直接投资4.2亿美元，年均增长23.9％。利用外资从传统的制造业拓展到基础产业、基础设施、高新技术以及第三产业。

(五)产业化步伐不断加快，支柱产业初步形成。农业产业化建设深入推进，建成了一批特色化、区域化、规模化的优势农产品和水产养殖生产基地。目前，全市拥有一定规模和水平的各类型农业产业化组织129个，龙头企业37家，其中省级龙头企业9家。电器机械、纺织服装、食品饮料、金银珠宝首饰工艺品和圣诞礼品等五大传统支柱产业不断发展壮大。形成了公平的服装、可塘的珠宝、碣石的圣诞礼品、梅陇的金银首饰工艺品、鹅埠的制鞋、海城的纺织、市区的电子信息和海产品深加工等专业生产基地。

(六)金融形势稳定，财税收入大幅增长。财税管理进一步加强，激励型财政政策得到落实，收入渠道不断拓宽，财政收入实现突破性增长。2006年全市实现地方财政一般预算收入9.27亿元，按可比口径比2003年翻了一番。其中，国税(不含海关代征)收入1.47亿元，地税收入3.89亿元，分别比2003年增长145％和98.5％。财政体制改革稳步推进，财政工作水平不断提高。金融形势平稳，2006年底金融机构各项存款余额164.35亿元，比2003年底增长63％。

(七)商贸流通活跃，第三产业快速发展。以现代流通业为主的新型服务业体系逐步健全，连锁经营、物流配送、大型超市、专卖店、电子商务等新的流通组织加快发展，民资和外资积极投向商贸流通领域，初步形成了多种所有制企业共同发展的良好局面。流通行业规范化管理得到加强，市场环境进一步改善。房地产业、旅游业、信息业等第三产业快速发展。

(八)和谐汕尾建设扎实推进，人民生活水平稳步提高。三年来，全市新增城镇就业6.6万人，转移农村劳动力就业11.7万人，城镇登记失业率控制在3.4％以内。社会保障体系不断完善，覆盖面进一步扩大，社保费征缴大幅度增长，企业离退休人员养老金和其它各项社保待遇按时足额发放。符合条件的城乡居民基本纳入最低生活保障范围；农村合作医疗覆盖面100％，参合人数159万人，参合率77.3％；全市80％行政村实现公路硬底化；解决了全市33.1万人饮水安全问题；认真落实农村免费义务教育政策，帮助3.61万农村困难子女解决入学难问题；完成危房改造3000多户。改造面积20多万平方米，帮助受灾群众重建家园4188户7291间，涉及人民群众切身利益的"一保五难"问题得到较好解决。社会主义新农村建设稳步推进，城乡居民收入逐年增加，2006年全市农民人均纯收入4008元，比2003年增长11.2％；城镇居民可支配收入8819元，比2003年增长26.9％。

政治社会局面稳定。社会治安综合治理进一步强化，各级公安机关深入开展严厉打击"双抢一盗"、凶杀贩毒、走私贩私等违法犯罪活动，刑事案件发案率明显下降，我市成为全省社会治安最好的地区之一。高度重视信访工作，全市信访量逐年下降，2004年度被评为全省信访工作先进集体。安全生产工作不断加强。创办"行风热线"，认真解决群众热点难点问题。特别是在省委、省政府的正确领导下，妥善处置了红海湾东洲"12.6"事件，维护了我市稳定发展的大局。

(九)精神文明建设成效显著，社会事业全面发展。保持共产党员先进性教育活动、"双融双建"活动和文明城市创建活动扎实开展。社会公德、职业道德、家庭美德、"爱国、守法、诚信、知礼"等主题教育活动不断深入。公民道德水平逐步上升，文明素质不断增强。城乡精神文明建设全面协调发展，全市有4个单位获得"全国文明单位"，2个村镇获得"全国文明村镇"的称号。军(警)民共建活动和双拥工作取得新成效。人民防空和民兵预备役工作取得新成绩。

教育事业有新发展。义务教育进一步巩固提高，高中阶段教育规模快速扩大。教育教学质量稳步提高。高等教育和成人教育稳步发展。342所学校危房得到改造，小学"两部制"教学问题基本解决，办学条件明显改善。科技工作取得新突破。三年来，全市新增专利申请569件，获国家授权348件，信利半导体有限公司OLED(有机发光平板显示技术)被国家科技部列为"863计划"。人才培养工程顺利推进。文化大市建设步伐加快。成功举办了"首届泛珠三角(广东汕尾)民间艺术节"、"纪念彭湃同志诞辰110周年活动"等一系列文化活动。正字戏、西秦戏、白字戏、皮影、滚地金龙等五个项目被国务院列为首批国家级非物质文化遗产，红宫红场被中宣部确定为全国爱国主义教育示范基地。卫生工作进一步加强。基本完成公共卫生三大体系建设任务，成功防范禽流感疫情，疾病防控工作成效显著。体育事业全面推进。全民健身运动蓬勃开展，竞技体育水平稳步提高。三年来，共获得全运会金牌3枚、全国锦标赛金牌7枚、省运会金牌18枚。计生、殡改工作取得新成效。人口与计划生育管理体系和服务体系不断完善，省下达的人口与计生工作目标顺利完成；殡改工作不断加强，火化率进一步提高。

物价、审计、国土、林业、环保、气象、残联、司法、台务、港澳、老龄、防震、广播电视、新闻出版、外事侨务、民族宗教、工会、共青团、妇女儿童、档案修志等工作全面推进。

(十)政府自身建设得到加强，民主法制建设稳步推进。各级 政府坚持向人大及其常委会报告工作，向政协通报情况，自觉接受监督。认真办理人大代表议案、建议、意见和政协委员提案，人民群众普遍关心的热点、难点问题得到较好解决。基层民主建设和村民自治工作进一步加强。各级政府勤政为民、廉洁自律的自觉性，依法行政能力、办事效率和服务水平进一步提高。行政审批制度、财政管理体制、投资体制、人事制度等改革深入推进，经营性用地出让、建筑市场、产权交易、政府采购等进一步规范，监察、纠风等工作取得新成效。

各位代表，过去的三年，是我市经济实力明显增强，社会事业全面进步，人民生活不断改善的三年。经过"十五"时期的快速发展和"十一五"时期开局之年的扎实工作，我市经济社会进入了新的快速发展时期，为全面建设小康社会奠定了坚实的基础。这是在市委的领导下，全市人民团结拼搏、艰苦奋斗的结果，也是人大、政协和社会各界关心、支持、帮助的结果。在此，我代表市政府，向全市人民，向各位人大代表、政协委员，向离退休老领导、老同志，向驻汕部队、武警官兵以及各界人士，向所有关心支持汕尾经济社会发展的港澳台同胞、海外侨胞和国内外友好人士，致以崇高的敬意和衷心的感谢!

回顾三年来的工作，我们深深体会到：必须树立发展的理念，坚持以科学发展观统领全局，狠抓发展第一要务不放松，勇于开拓创新，不断优化经济结构，保持经济又好又快增长；必须营造发展的氛围，坚持实事求是、真抓实干、团结奋进，努力提高各级干部的工作水平和工作实效性，最大限度地调动全市人民的积极性、主动性和创造性，形成加快发展的强大合力，确保各项工作任务落实到位；必须强化发展的措施，切实抓好招商引资工作，大力发展县域经济和民营经济，认真抓好生产力骨干项目、基础设施重点项目建设，努力推进我市实现跨越发展；必须筑牢发展的根基，高度重视和谐社会建设，狠抓薄弱环节，切实维护好社会稳定，坚持以人为本，把实现好、维护好、发展好人民群众的根本利益作为政府工作的出发点和落脚点，千方百计为群众办实事、办好事，让人民群众在改革和发展中得到更多的实惠。

与此同时，我们必须清醒地看到经济社会发展中仍存在一些问题和困难，主要是：经济综合实力弱、基础差、总量小，与经济发达地区的差距较大；经济结构不够合理，缺乏支柱产业，财政实力较弱；县域经济发展不平衡，农民增收缓慢；就业和社会保障任务重，压力大；社会公共服务体系不够健全，法制环境有待进一步完善；各项工作水平和实效性尚需提高；计生、殡改、社会治安等方面还存在一些不容忽视的问题。我们必须高度重视，并在今后的工作中采取有力措施，认真加以解决。

今后五年的指导思想和目标任务

　　今后五年，是我市进一步落实科学发展观，全面建设小康社会的关键时期，也是实现省委、省政府提出"三年打基础、五年大变化、十年大发展"目标要求的关键时期。当前，我们面临十分难得的发展机遇，也具备了跨越发展的坚实基础和有利条件。我们必须认清形势，抢抓机遇，凝聚力量，乘势而上，努力推进汕尾更好更快地发展。

今后五年政府工作的指导思想是：坚持以邓小平理论和"三个代表"重要思想为指导，以科学发展观统领经济社会发展全局，认真贯彻落实党的十六大、省九次党代会以来的一系列方针政策和粤东会议、市五次党代会精神，以"三年打基础、五年大变化、十年大发展"，尽快赶上全省总体发展水平为总要求，按照经济、政治、文化、社会建设"四位一体"的总体布局，狠抓发展第一要务，落实稳定第一责任，进一步落实跨越发展战略，大力发展"三基地三经济带"，全面实施"八大工程"，切实加强民主法制建设，加快推进和谐社会建设，努力把汕尾建设成为工业、旅游业和海洋产业为主导的现代化滨海新城。

今后五年的奋斗目标是：抢抓新机遇，推进新跨越，共建新汕尾，努力打造活力汕尾、人文汕尾、和谐汕尾、清新汕尾。

经济发展迈上新台阶，经济结构得到优化，城乡居民收入明显增加，人口素质明显提高，物质文化生活更加丰富，社会保障体系更加健全，可持续发展能力进一步增强，社会更加和谐稳定。力争地区生产总值年均增长18％；三次产业结构调整为13.1:54.1:32.8；工业总产值年均增长26％；社会消费品零售总额年均增长18％；全社会固定资产投资年均增长28％；财政一般预算收入年均增长25％；外商直接投资年均增长27％；外贸出口总额年均增长30％；城镇居民人均可支配收入年均增长9％；农民人均纯收入年均增长6％；城镇登记失业率控制在3.5％以内；人口自然增长率控制在7.3&permil;以内；每万元GDP能耗控制在0.51吨标煤以内。

实现今后五年的奋斗目标，必须在"六个着力"上下功夫：

(一)着力落实科学发展观。始终坚持以科学发展观统领经济社会发展全局，狠抓发展第一要务，进一步落实又好又快的发展要求，实现速度与质量、效益、结构相统一，经济发展与人口、资源、环境相协调。

(二)着力增强综合经济实力。坚持以经济建设为中心，围绕省委、省政府提出的"三年打基础、五年大变化、十年大发展"的目标要求，打造电子信息、电力能源、石化工业等三大产业基地；发展蓝色产业经济带、绿色产业经济带、特色旅游产业经济带等三大经济带。

(三)着力增强发展动力。深化行政管理体制、事业单位和国有企业改革，依法加强监管，盘活国有资产，推动金融改革与发展，健全现代市场体系。实施自主创新战略，加强资金扶持和政策引导，促进重点领域自主创新和科技成果转化，鼓励企业提高自主创新能力，创造自主品牌，大幅度提高经济发展的科技贡献率。优化利用外资和出口商品、出口市场、贸易方式结构，更加主动承接国际、国内产业转移，推动内外源型经济相互促进、协调发展。按照适度超前的要求，加大重点项目建设力度，着重完善交通、能源、水利等基础设施，发挥重点项目对经济发展的拉动力。

(四)着力促进城乡和县域协调发展。统筹城乡公共基础设施建设，加强农村规划、建设和管理，以农业产业化为基础，工业化为龙头，城镇化为载体，发展壮大县域经济，加强县城、中心镇和专业镇建设，不断增强城镇对农村的辐射带动能力。鼓励和引导城镇社会事业向农村延伸，努力建设社会主义新农村，促进城乡统筹发展。

(五)着力推进文化大市建设。大力培育现代公民，造就现代文明，构建现代社会，推动精神文明建设上新台阶。优先发展教育事业，改善中小学办学条件，促进义务教育均衡化、高中教育普及化、职业技术教育特色化、高等教育大众化，不断推进教育现代化。改革文化体制，完善促进文化发展的政策措施，发展文化事业和文化产业，推动文化与经济进一步融合。实施人才培养工程，建设一支结构合理、创新力强的人才队伍。

(六)着力建设和谐汕尾。全面开展"平安汕尾"建设，致力营造公平正义的法治环境、诚信守法的市场环境、规范高效的政务环境、文明和谐的人文环境、安全有序的治安环境。坚持执政为民，认真解决人民群众切身利益问题。切实关心和改善人民生活，不断增加城乡居民收入。实施积极的就业政策，努力促进就业和再就业。完善社会保障制度，进一步扩大社会保险覆盖面。完善医疗卫生体系，进一步落实农村合作医疗制度。加大力度解决困难群众生产生活问题。繁荣体育事业。突出抓好计生、殡改和安全生产工作。大力实施绿色汕尾战略，扎实推进社会节能降耗，节约利用资源，发展循环经济，保护生态环境，控制污染物排放总量，努力建设资源节约型、环境友好型社会。

2007年工作安排

　　2007年是我市贯彻落实粤东会议精神的第一年，也是实施"十一五"规划的第二年。做好今年各项工作意义重大、责任重大。我们要坚持以科学发展观为指导，深入贯彻中央经济工作会议、省九届十次全会、粤东会议和市五次党代会精神，按照"三年打基础、五年大变化、十年大发展"的总体要求和市委的战略部署，统筹全局、改革创新、注重和谐、关注民生，加快生产力骨干项目和基础设施建设步伐，全面推进社会主义新农村建设，努力构建和谐社会，实现经济社会又好又快发展。

今年我市经济社会发展的预期目标是：地区生产总值增长18％；农业总产值增长6％；工业总产值增长29％，规模以上工业产值增长35％；第三产业增加值增长16％；社会消费品零售总额增长18％；全社会固定资产投资增长30％；居民消费价格涨幅控制在3％左右；地方财政一般预算收入增长25％；外商直接投资增长30％；外贸出口总额增长30％；城镇登记失业率控制在3.5％以内；农民人均纯收入增长6％，城镇居民可支配收入增长9％；人口自然增长率控制在6.76％以内；每万元GDP能耗控制在0.534吨标煤以内。

为此，必须大力推进十项工作：

(一)大力推进"三大基地"建设，壮大发展实力

打造电子信息产业基地。认真实施电子信息产业基地发展规划，以提升我市电子信息产业整体区域竞争力为目标，建设好市区显示器园区(南园)、电子元件园区(北园)、产业拓展园区(东园)三大园区。以信利、德昌电子信息企业为龙头，大力发展高新技术领域的电子信息产业，建设以TFFT、二极管和三极管等产品为重点的电子信息产业基地，使之成为我市的工业支柱产业。

打造电力能源产业基地。重点推进汕尾发电厂、陆丰核电站、陆丰甲湖、甲东风电场、国投大华发电厂、500千伏汕尾输电工程配套项目、500千伏汕尾发电厂送出工程以及滨海、河口110千伏输变电工程等项目建设，努力把汕尾建设成为全省乃至全国重要的电力生产基地。

打造石油化工产业基地。以在建的海丰小漠华城能源有限公司为启动项目，大力推进海丰石化项目的建设，认真抓好陆丰石化工业园区项目的前期工作，配合好粤东成品油管道(汕尾段)建设，积极引进石油化工中下游产品，大力发展石化工业，努力把石油化工培植成为我市新兴支柱产业。

(二)大力推进"三大经济带"建设，优化发展布局

发展蓝色产业经济带。尽快完成海洋功能区划的编制工作。以建设汕尾国家级中心渔港为契机，加大渔港基础设施建设投入，推动我市现代渔港经济区建设。认真做好临港工业区的规划，大力发展临港工业，重点发展装备制造、海洋生物、电子信息、高新技术和港口物流等产业。大力发展海洋捕捞业，重点发展水产养殖业和水产品深加工业，积极开发应用推广养殖新技术，加快发展水产特色品牌产品。加快无公害水产养殖、水产养殖种苗、水生生物资源保护、渔业保障体系建设以及海岛资源开发等五个项目建设，大力发展罗非鱼、水产良种、对虾、深水抗风浪网箱、海马、牡蛎等六大养殖基地，努力打造海洋经济支柱产业。

发展绿色产业经济带。在确保粮食生产稳步发展的同时，加快发展效益农业和现代农业。推进特色农产品生产和加工基地的建设，重点建设综合性种养示范项目和陆河生宝橄榄良种繁育基地、陆丰黄牛综合养殖基地、海丰梅陇、小漠综合种养基地等一批现代农业示范基地，积极争取省粤东粮食储备库落户我市。培育发展一批国家、省、市级农业龙头企业和专业合作社组织，巩固壮大我市9家省级农业龙头企业，扶持培育国泰食品有限公司、五丰水产食品有限公司、海丰食品企业集团、绿洲农贸有限公司、顺和成林纸综合开发有限公司等5家农业企业为省级农业龙头企业。全面推广"公司+农户+基地"经营方式，不断增加农民收入。充分利用省安排的500万元启动资金，高起点建设农业现代化示范区。积极引导龙头企业参与农业现代化示范区建设。完善农产品标准体系、质量检测体系和动植物防疫体系建设，加强农产品质量安全管理。

发展特色旅游产业经济带。充分发挥我市环境、区位和资源优势，大力发展旅游休闲度假业。完善旅游规划，整合旅游资源，以"红、蓝、绿、古"四色旅游为主题，加强星级酒店等旅游基础设施建设，丰富旅游文化内涵，制定精品旅游线路，提升旅游服务质量，打响汕尾特色旅游品牌，推进旅游产业化进程。加快红宫、红场、凤山祖庙、滨海景区建设，着力推进南万红椎林和公平、大湖两个候鸟省级自然保护区，以及生态园林度假基地等景区的开发建设，培育一批有较强竞争力的旅游业龙头企业。努力创建中国优秀旅游城市，把汕尾建设成为大珠三角地区旅游度假的东花园。

(三)大力推进基础设施建设，增强发展后劲

加快以完善大交通为重点的基础设施建设。进一步强化交通设施建设。公路建设方面，重点抓好国道324线汕尾路段大修和改线工程、汕尾至遮浪一级公路、S242线西闸至埔边、市城区凤飞山至捷胜公路、S242线公平段改线工程等公路建设。抓紧镇通镇和镇通建制村路面硬底化公路建设。港口建设方面，加快汕尾新港区的规划建设，使其与海丰小漠石化码头、汕尾港航道扩建工程及马宫港、碣石港、乌坎港等港口码头建设同步推进，形成粤东新港口群。铁路及高速公路建设方面，认真配合做好广西玉林至福建漳州高速公路汕尾路段的工可及设计工作，加快推进汕尾至江西瑞金高速公路汕尾路段的规划前期工作，全面动工建设厦深铁路汕尾段。

加快以完善城市基本功能为重点的城市基础设施建设。

按照高起点规划、高标准建设、高水平管理的要求，加强城市规划、建设和管理，建设区域中心城市。围绕市区品清湖，实施城市"东扩"战略，拓展新城区，改造老城区，加快推进市区建设，进一步完善和拓展市区作为区域中心的城市功能，不断增强城市辐射带动作用。抓紧完成全市土地利用总体规划的修编和控制性详细规划的编制工作，推动品清湖沿岸综合开发建设项目的落实。以市区配套设施建设为重点，加快市区道路建设步伐，推进城市天然气管道等市政设施建设，抓紧规划建设体育场、体育馆、群艺馆、文化馆等公共活动设施，增强城市服务功能。加快发展物业管理，大力整治脏乱差，加快城市景观建设，改善城市环境，提升城市品位。

加强水利基础设施建设。重点建设市区、海丰、陆丰、陆河、红海湾等5宗防洪(潮)工程；南告等7座中型水库除险加固工程；陆丰乌坎海堤等9座堤围达标加固工程；公平等7个灌区改造工程；改造乌坎、西闸2座大型出海口水闸。实施省人大小型水库除险加固、机电排灌改造、水库移民安置三大议案工程。

(四)大力推进外源型经济发展，提高利用外资水平

加大招商引资力度。继续实行招商引资领导分工责任制，创新招商引资方式，拓宽招商引资渠道，充分发挥企业在招商引资中的主体作用，构筑"政府带动、企业主动、社会互动"的全民招商引资联动机制，切实提高利用外资质量和水平。充分发挥我市毗邻港澳、侨胞众多的优势，做好做活"侨"字文章，着力办好"侨博会"，组织参加粤港经贸合作交流会等境内外招商洽谈会，抓好高新技术产业开发区等重点园区的招商引资工作，做到以侨引商，以情引商，以商引商，力争吸收外资取得新突破。

积极扩大外贸出口。积极扩大高新技术产品出口，继续组织好我市传统特色产品、深加工农副产品、海水产品等产品出口。大力扶持民营企业出口创汇，积极开拓国际市场。鼓励来料加工转进料加工，促进加工贸易转型升级，推动一般贸易发展壮大，努力提高我市出口产品的创汇能力。培育建立服装、制鞋、金银珠宝首饰工艺品、圣诞礼品、水果加工、水产品加工等一批出口基地，增强市场竞争力。在巩固港澳台、美国、加拿大、日本、欧盟等传统出口市场的基础上，积极拓展俄罗斯、南美、非洲、中东和东盟等有增长潜力的出口市场，实现出口市场多元化。优化汕尾大通关环境，努力扩大外贸进出口规模。

扩大利用外资规模。积极引导外资投向我市的工业发展项目，着力引进大财团、大企业到我市投资置业，提升我市的工业规模和水平。支持鼓励外商增资扩产，加大资金投入规模，促进我市工业发展上新台阶。重点做好信利半导体TFT、德昌电子(三期)等项目增资扩产工作。大力培育主业突出、具有自主知识产权和核心竞争力的强势企业，做大做强一批大企业和企业集团，使之成为产业集群的核心，充分发挥龙头带动作用。

优化投资环境。提高服务质量，完善优惠政策，依法保护投资者合法权益，营造良好的投资环境。推进市行政服务中心建设，增强引资吸引力和竞争力，营造"人人都是招商主体、人人都是投资环境、人人都有开放责任、人人都支持引进外资"的良好氛围。加强对外经济技术合作与交流，鼓励和支持有条件的企业开展对外投资，发展境外加工贸易和劳务输出，推进企业跨国经营。

抓好园区建设。对现有工业园区进行科学规划和优化整合，高效、合理利用土地资源。完善基础设施配套，使园区成为集聚产业功能、资金技术、巨大产能和增长潜能的重要经济载体。重点抓好红海湾经济开发试验区、陆丰东海经济开发试验区、海丰老区经济开发试验区、陆河工业园、陆丰星都经济开发区的建设，把建设工业园区和发展产业集群结合起来，构筑工业发展大平台。认真做好与东莞(大朗)、深圳(水田)共建产业转移园区的工作。

(五)大力推进内源型经济发展，做大经济总量

加快重点项目建设。根据省委、省政府促进粤东发展项目规划和汕尾市"十一五"重点项目规划，结合我市的发展需要，拟实施408个重点项目，计划总投资1358.1亿元，"十一五"期间投资652.3亿元，其中列入粤东发展规划的重点项目58项，总投资737.3亿元，"十一五"期间投资311.7亿元。2007年初步确定全市重点建设项目27项，总投资183.1亿元，年度计划投资50.4亿元。重点抓好汕尾发电厂、陆丰核电站、厦深铁路(汕尾段)、信利TFT等一批重点项目，市区要重点抓好环品清湖沿岸堤路项目、海陆科技、华师附中和碧桂园、万福、华策等房地产开发项目的建设。加强项目的申报和储备，争取多一些项目进入国家、省的计划安排。

促进民营经济再上新台阶。认真落实鼓励扶持民营经济发展的政策措施，扩大准入领域，降低准入门槛，拓展创业范围，积极引导民间资本投入主导产业和支柱产业，推动民营经济大发展。推进面向民营企业和中小企业的信用担保体系建设，为民营企业解决融资难等实际困难。鼓励有条件的民营企业通过兼并、收购、联合等方式优化组织结构，发展成为主业突出、竞争力强的大公司大集团。引导民营经济向科技型、外向型、规模效益型方向发展。积极支持建立行业商会，增强行业自我发展的能力。引导民营企业依法经营，全面提高民营经济素质和水平。

增强自主创新能力。实施"促进粤东加快发展科技行动计划"，强化企业在自主创新中的主体地位，鼓励企业进行技术改造、技术创新和新产品的开发，开展产学研结合，增强自主创新能力。突出抓好重点领域的科技攻关，力争在电子技术、养殖技术、新材料等领域有新突破。抓好公平、可塘、碣石、东海、东坑等五个省级技术创新试点镇和公平镇产业集群升级示范区的技术创新。扶持国家和省火炬计划特色产业基地建设。加强产业关键共性技术平台建设，设立科技型中小企业创新基金，推进传统产业技术升级和信息化改造。实施名牌带动战略，加大对假冒伪劣商品的打击力度，培育和发展具有自主知识产权的名牌产品，力争新增10个以上省级名牌产品。深化国有企业改革，健全国有资产监督管理体制，确保国有资产保值增值。以专业镇、中心镇为载体，以高新技术和先进适用技术改造提升电器机械、纺织服装、食品饮料、金银珠宝首饰工艺品和圣诞礼品等五大传统工业产业。

加强资源节约和生态环境保护。加强资源节约与合理高效利用，大力发展循环经济。积极做好节能、节水、节材工作，大力发展优质、低耗产品，推行清洁生产，发展节约型产业。按照权责一致的原则，落实市、县政府土地管理责任，盘活存量土地，提高土地利用效率。加快城镇生活污水和垃圾处理设施建设，推进治污保洁和螺河等河流整治工程。继续开展环保执法专项行动。

扶持发展新兴服务业。鼓励发展商务、物流、通讯、仓储、会展等现代服务业，引导发展社区服务、物业管理、文化娱乐、体育健身、医疗保健等公共服务，积极发展信息咨询、法律、会计、审计、劳务等中介服务业。建设农产品、水产品和工艺品等专业批发市场，推动现代物流发展。加快发展旅游房地产业，推进信息产业建设。积极扶持金融、保险等产业的发展。

(六)大力推进区域协调发展，加快城镇化进程

大力发展县域经济。按照全市产业结构和生产力空间布局规划，充分发挥各县(市、区)的比较优势，大力促进区域平衡发展，壮大县域经济实力。海丰要继续发挥排头兵作用，加强中心镇、专业镇建设，努力建设经济强县；市城区要依托市区优势，发展城市经济，努力建设现代化滨海新城；陆丰要以核电站上马建设为契机，迎难而上，奋起直追，努力建设工业化城市；陆河要发挥山区资源优势，发展特色产业和生态农业，努力建设经济发达的新兴县；红海湾开发区要充分发挥汕尾发电厂带动效应和滨海优势，努力建设临港工业区和旅游度假区；华侨管理区要发挥侨区优势，努力建设现代新侨区。积极实施"强镇富民"战略，推动"一镇一品"、"一村一品"建设，形成"专、精、特、新"的产业优势。继续扩大县级经济管理权限，切实加大对陆丰等地区的扶持力度，进一步完善县级镇级党政领导实绩考核制度，充分调动发展县域经济的积极性和主动性，增强县域经济发展活力。

推进城乡统筹发展。按照"省管县、市管镇、县管村"的原则，抓好城乡发展规划，逐步形成以中心城市和中心镇为依托，以骨干公路沿线为主轴的集约发展态势，建立起组合有序、优势互补、协调发展的城镇新体系。推进城镇基础设施建设，加快城镇化进程。加强中心镇的规划、建设和管理，加大对中心镇的基础设施建设投入，优化完善服务功能，改善中心镇的生产生活环境，增强城镇集聚力和带动农村发展的能力，推动农村富余劳动力向非农产业和城镇转移。加强城乡生态环境建设和文明建设，争创国家"文明城市"。从2007年起，各县(市、区)抓好创建国家卫生城市、中国优秀旅游城市的各项前期工作，争取2008年创建国家卫生城市和中国优秀旅游城市。大力推动城镇建设投资主体多元化，走以城养城、以城建城、以城兴城的市场化之路。

(七)大力推进财税工作，不断壮大财政实力

培植壮大财源。千方百计扩大财税收入规模，执行稳健的财政政策，加快公共财政体制建设步伐。继续实施《汕尾市县(市、区)经济社会发展考核办法》、《汕尾市乡镇党政主要领导工作实绩考核(试行)办法》、《汕尾市纳税大户奖励办法》。充分利用各种财税政策，培育税收骨干企业，改善财政收入结构，培植新的税收增长点，努力增加财政收入。充分发挥和利用好省扶持我市四年每年5000万元，以及支持15个市2007、2008每年各1亿元的产业发展引导资金，推动产业优化升级，加快基础设施建设和支柱产业发展。

强化征收管理。坚持收入分析和收入组织工作制度，以及财税收入完成情况联席会议制度。坚持依法治税，实行领导分工抓收入，强化监督检查，挖掘潜力，堵塞漏洞。优化税收法治环境，完善纳税信用体系建设。加大对重点税源、重点行业的征收、稽查力度，完善非税收入管理制度，规范土地、房地产等非税收入管理，加强行政收费、公共资源有偿收入等收缴，坚决打击各种偷税、逃税、抗税行为，切实提高征管质量，确保税收如期足额入库。

优化支出结构。坚持"量入为出、收支平衡、略有节余"的理财原则，推进部门预算改革，强化国库集中收付、政府采购制度和"收支两条线"管理。整合财政资源，统筹使用和调度各类资金，按照"保重点、压一般"的原则，继续调整和优化支出结构，确保人员工资、教育、公共卫生、再就业、社保、济贫解困等重点支出。

防范化解财政和地方金融风险。健全财政抗风险应急预警机制，增强财政调控能力。加强对财政资金使用的监督检查和追踪问效工作，保证财政资金稳定、安全、高效运转。加强社会信用环境建设，加大债务清理和催收力度，继续做好资产处置和城市信用社退市工作，严厉打击逃废金融债务行为，防范化解金融风险，创造金融企业支持地方经济建设的良好环境。

(八)大力推进"三农"工作，努力建设社会主义新农村

不断增加农民收入。建立健全农业农村服务体系，扎实推进"万村千乡"市场工程，办好农民专业合作社，大力提高农民的组织化程度，增强农业龙头企业在农产品生产、加工、销售的主导作用，提高农产品的市场竞争能力。支持高收入户二次创业，带动低收入户脱贫致富。加强劳动力职业技术培训，扎实推进"百万农村青年技能培训工程"，加快农村富余劳动力转移，增加农民非农收入。继续组织机关干部进村入户帮扶创业，掀起农民致富奔康热潮。以巩固税费改革成果为重点，全面推进农村各项改革，进一步完善农民增收减负的长效机制。

切实改善农村生产生活条件。抓好村级规划编制工作，加大农村道路、公共设施和文化设施建设的投入，加大整治力度，改善村容镇貌。加强农田水利设施建设和城乡水利减灾防灾工程建设，继续推进农村"一保五难"工作。大力推进农村危房改造和安居房工程建设。以创建生态文明村镇为切入点，认真抓好农村"五改"工作，改善农业农村生态环境。多方筹集资金，建设农村饮水工程，解决农村饮水安全问题。大力兴办农村公共事业，加快发展农村教育、卫生和文化等事业。推进农村社会保障体系建设，扩大新型农村合作医疗参合率。加强镇卫生院和村卫生所的建设，改善农村预防保健和医疗救治条件，完善疫情应急预警和处置机制。加快林业生态建设步伐，提高森林覆盖率。

强化农村民主管理。进一步加强农村基层组织建设，健全村民自治机制，完善村务公开和民主议事制度。深入开展农村普法教育，增强农民法制观念，提高农民依法行使权利和履行义务的自觉性，促进农村民主化、制度化和法制化建设。

(九)大力推进各项事业建设，确保社会和谐稳定

强化社会保障工作。进一步完善社会保障体系。以非公有制企业为重点，扩大社会保险覆盖面。加强社会保障基金的征缴和管理，提高社会保障水平。全面开展退役士兵免费职业技能培训，做好军转干部、退伍军人以及企业下岗职工安置、分流工作。关心残疾人事业，落实城乡最低生活保障制度。建立和完善以生产、生活、教育、医疗、住房、法制等为主要内容的社会援助和救助体系，切实解决困难家庭就医难、入学难、住房难等问题，广泛开展为困难群众送温暖、献爱心活动，逐步形成全社会帮弱、助残、扶贫、济困的良好氛围。实施扩大与促进就业工程，重点帮助城镇"零就业"家庭，农村贫困家庭等困难群体实现就业。

全力维护社会稳定。牢固树立"维护稳定是第一责任"的意识，努力创建"平安汕尾"。全面落实社会治安综合治理责任制，健全群防群治防控体系，精心组织开展各种严打专项整治斗争，严厉打击黑恶势力和刑事犯罪，坚决遏制"黄赌毒"，构建反走私打防体系，维护良好的治安秩序。加强劳动保障监察，及时查处拖欠工资等劳动违法行为，维护农民工合法权益。切实抓好信访和社会调解工作，及时将社会矛盾化解在萌芽状态。认真落实安全生产责任制，预防和减少重特大安全事故的发生。加强消防、道路安全、水上交通和食品药品安全工作，消除各种安全隐患。做好防灾减灾工作。

切实加强精神文明建设。深入开展"爱国、守法、诚信、知礼"现代公民教育活动，切实加强以社会主义荣辱观为主要内容的社会主义荣辱观教育，努力营造和睦相助、友爱向善、谅解宽容的人文环境。继续加强和改进未成年人思想道德建设。大力弘扬以改革创新为核心的时代精神。推行科学、文明、健康的生活方式，坚决反对封建迷信。积极开展创建"文明村镇、文明社区、文明行业"系列活动，广泛开展社会公德、职业道德、家庭美德教育活动，促进群众性精神文明创建活动上新水平，提高市民整体素质，努力培养勤劳致富、守法经营、踏实肯干、文明守信的现代新型公民。加强社会主义法制建设，全面开展"五五普法"教育，推进依法行政，促进司法公正。

优先发展教育事业。大力发展职业技能培训，建设好一个职业技术教育公共实训中心和一个高技能人才培训交流基地，尽快完成市技工学校的扩建工作。大力发展职业技术教育，力争2007年5所示范性职校每年招生规模1000人以上，其他职校600人以上，汕尾职业技术学院全日制在校生5000人以上，市技工学校在校生1500人以上。巩固和提高"普九"成果，增强高中自主办学能力，加快普及高中阶段教育，在继续推进7所示范性普通高中建设的基础上，每个县(市、区)新建、扩建1所以上普通高中学校；加快华师附中汕尾分校的建设步伐，确保2008年秋季正式招生；加大力度推进骨干示范性职校建设。努力改善农村中小学办学条件，加快危房校舍改造，2007年底前全面完成C、D级学校危房改造。做好免除农村中小学生课本费工作。科学配置教育资源，促进教育均衡、协调、优质、高效发展。加强师德师风建设，推进素质教育，提高教育教学质量，不断增强我市教育的综合实力。优化人才工作体制和社会环境，培养、吸引和用好创新型专业人才和高技能人才，为促进经济社会发展提供强有力的人才支撑。

推进文化大市建设。深化文化体制改革。加强对文化的发掘和研究，打造独具海陆丰特色的文化品牌。增强海陆丰文化的开放性、兼容性和时代性。抓好重点文化遗产和优秀民俗民间文化的保护和开发利用。努力繁荣文艺创作，为群众提供积极向上、丰富多彩的精神食粮。大力发展文化产业，培育和规范文化市场体系。加强文化基础设施建设，丰富群众精神文化生活。

全面发展社会事业。完善公共卫生服务体系，提高预防保健和疾病控制水平。建立健全农村三级卫生服务网络和以社区卫生服务为基础的城市卫生体系。完善食品药品安全保障体系，实施食品药品放心工程。广泛开展全民健身运动，不断提高全民健康水平。依法加强人口与计划生育工作，创新人口与计划生育管理机制，促进人口与计划生育工作上新水平。坚持移风易俗，大力推行火葬。认真实施妇女儿童发展纲要，加强对未成年人和妇女的权益保护。加强军政军民团结，着力做好双拥工作，积极创建双拥模范城。建立完善国防动员体系，进一步提高民兵预备役建设水平。促进物价、审计、监察、统计、人事、人防、气象、防震、台务、港澳、老龄、广播电视、新闻出版、民族宗教、外事侨务、妇女儿童、档案修志等工作全面发展。

(十)大力推进政府自身建设，提高政府行政效能

切实转变政府职能。认真履行经济调节和市场监管职能，强化社会管理和公共服务。建立健全重大事项社会公示、听证和专家咨询等制度，畅通民意沟通渠道，扩大公众的知情权、参与权、决策权和监督权，提高政府决策的科学性、民主性和透明度。大力发展电子政务，优化行政服务流程。创新政府管理方式，进一步理顺政府与企业、市场、社会的关系。

切实加强作风建设。树立和实践正确的政绩观和荣誉观，求真务实，真抓实干，多做群众急需、作用长远的事。推行工作目标项目化，项目推进责任化，加强督查，严格考核。继续推进行政审批制度改革，简化审批程序，规范审批行为。大力精简会议、文件，严格控制检查评比，提高行政效率。

切实推进依法行政。主动接受市人大及其常委会的法律监督和工作监督，接受市政协的民主监督，加强与民主党派、工商联、人民团体和无党派人士的联系。认真执行市人大决议，积极办好人大代表议案和政协委员提案。认真贯彻实施《全面推进依法行政实施纲要》，严格行政执法执纪行为，强化行政执法责任考核和责任追究。落实政府规范性文件统一审查制度。

切实加强廉政建设。全面推进政务公开，完善经营性用地出让、建筑市场、产权交易、政府采购等招投标制度，坚决查处违法违纪行为，进一步健全惩治和预防腐败体系，从源头上防治腐败。加强法律、行政、社会、舆论等监督，抓好行风评议工作。认真实施国家公务员法，努力建设一支政治坚定、业务精通、清正廉洁、作风优良的高素质公务员队伍。

各位代表：粤东会议为我市加快发展吹响了号角，我市正面临着本世纪初一次难得的发展机遇。让我们更加紧密地团结在以胡锦涛同志为总书记的党中央周围，高举邓小平理论和"三个代表"重要思想伟大旗帜，坚持以科学发展观统领经济社会发展全局，在市委的领导下，认真落实粤东会议和市五次党代会精神，紧紧围绕"三年打基础，五年大变化，十年大发展"的目标要求，大力实施"三基地三经济带"和"八大工程"发展战略，振奋精神、抢抓机遇、真抓实干、狠抓落实，为把汕尾建设成为工业、旅游业和海洋产业为主导的现代化滨海新城而努力奋斗!
